# Generative Design and Integrated 3D Printing Manufacture of Cross Joints

**DOI:** 10.3390/ma15144753

**Published:** 2022-07-07

**Authors:** Leyu Han, Wenfeng Du, Zhuang Xia, Boqing Gao, Mijia Yang

**Affiliations:** 1 Institute of Steel and Spatial Structures, College of Civil Engineering and Architecture, Henan University, Kaifeng 475004, China; hanleyu1011@outlook.com (L.H.); xz1343331527@outlook.com (Z.X.); 2 Henan Provincial Research Center of Engineering Technology on Assembly Buildings, Kaifeng 475004, China; 3 Department of Civil Engineering, Zhejiang University, Hangzhou 310058, China; bqgao@zju.edu.cn; 4 Department of Civil and Environmental Engineering, North Dakota State University, Fargo, ND 58102, USA; mijia.yang@ndsu.edu

**Keywords:** cross joints, generative design, 3D printing, numerical analysis

## Abstract

The integrated process of design and fabrication is invariably of particular interest and important to improve the quality and reduce the production cycle for structural joints, which are key components for connecting members and transferring loads in structural systems. In this work, using the generative design method, a pioneering idea was successfully realized to attain a reasonable configuration of the cross joints, which was then consecutively manufactured using 3D printing technology. Firstly, the initial model and generation conditions of a cross joint were constructed by the machine learning-based generative design algorithm, and hundreds of models were automatically generated. Then, based on the design objective and cost index of the cross joint, three representative joints were selected for further numerical analysis to verify the advantages of generative design. Finally, 3D printing was utilized to produce generative joints; the influences of printing parameters on the quality of 3D printing are further discussed in this paper. The results show that the cross joints from the generative design method have varied and innovative configurations and the best static behaviors. 3D printing technology can enhance the accuracy of cross joint fabrication. It is viable to utilize the integrated process of generative design and 3D printing to design and manufacture cross joints.

## 1. Introduction

Joints are significant components for connecting members and transferring loads in various types of rod structural systems [1,2]. The cross joint has become one of the most commonly applied joint forms due to its simple construction and clear force transmission path [3]. However, conventional design and manufacturing methods are still used for the cross joints in existing projects, leading to falling behind the engineering practice requirements, such as unreasonable configuration, long cycle, and a high cost of joints [4]. Furthermore, the traditional design and fabrication stage of cross joints is usually independent. The manufacturability of the model is not considered in the design stage, which easily makes the designed joint scheme unable to be produced by traditional manufacturing processes [5]. Therefore, to pursue innovative ideas for solving the above problems, it is first necessary to summarize the current situation and development direction of the design and manufacturing technology.

**Figure 1:**
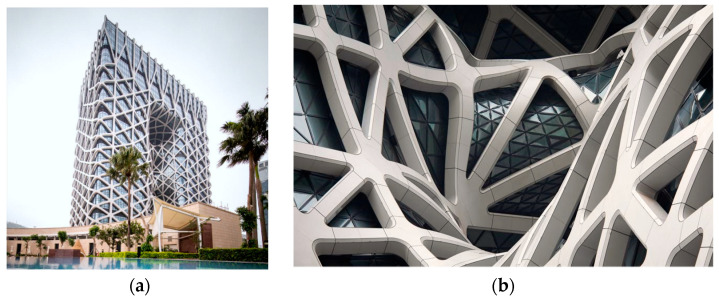
The Morpheus Hotel in Macau: (**a**) overall shape of building, (**b**) various joint configurations.

On the one hand, the design of cross joints still mainly depends on the engineers’ experience. Engineers first propose a preliminary joint model based on the conceptual design, and then the mechanical performances of the initial joints are calculated and analyzed in detail by finite element (FE) technology. Finally, the model’s adjustment is conducted according to the computation results. The above process is generally cyclic and repeated several times, and model testing must sometimes be carried out. Therefore, the traditional design method based on the engineers’ experience has a long cycle time and high energy consumption [6]. In particular, when the types and numbers of joints in an entire structure are substantial, such as in the Morpheus Hotel in Macau, as shown in Figure 1, it is almost impossible to complete the design assignment by the traditional method.

In recent years, the appearance of modern buildings is progressively complex, and it is difficult to design rational joint configurations by relying purely on the engineers’ experience. For structural safety, engineers normally assign more material to cross joints, which easily results in the design problems of excessive mass and insufficient geometric optimization. In response to the above problems, many scholars have conducted optimization design research on joints with the help of topology optimization methods [7,8,9], such as Zhang et al. [4], who proposed a topology optimization method of substructure based on bionics and used this method to attain the optimal model of a cross joint. From the current research status, topology optimization is a feasible method for the optimization design of structures. Nonetheless, the existing topology optimization method is only executed within the framework of the established optimized design area, which restricts the design exploration space. Moreover, topology optimization can only obtain a single outcome rather than several alternatives. If the topological convergence solution does not satisfy the aesthetic or other property requirements, a significant amount of manual adjustment work is required [6].

With the rapid development of artificial intelligence technology, the design of joints can be achieved by introducing more automatic and efficient methods. Generative design is a modern approach to comprehensive design schemes that explores the entire design space to achieve purposes such as minimal structural compliance, given a set of functional requirements and constraints based on geometry, material specifications, and fabrication considerations [10,11,12]. According to Krish [13], the concept of generative design was first proposed by Frazer in the 1970s. In 1989, with the advent of parametric CAD tools, generative design was further studied [14]. In 1997, Bentley and Wakefield [15] developed and optimized the first generation of generative design systems based on genetic algorithms. Then, some representative generative design methods were born, such as cellular automata, L-systems, and swarm systems [16]. However, there has been no substantial breakthrough. Since the rapid development and popularization of a new round of intelligent automation technology and optimization algorithms, generative design has achieved unprecedented potential and has been seriously studied and applied in certain fields [17]. For example, Gulanová et al. [18] proposed a surface-based the part generative engineering design method, along with its general application. In 2019, Oh et al. [19] built a generative design framework based on deep learning algorithms, which integrated topology optimization and generative adversarial networks in an iterative way. Ge [20] utilized a generative design for the lower limb structure of a biped robot and obtained hundreds of new design alternatives. Bright et al. [21] carried out generative design on a helicopter frame and gained a derivative frame model with better fracture and deformation resistance. Wu et al. [22] used the generative design method to optimize the roller seat of a wind turbine blade turnover machine. The generative roller seat not only guaranteed strength and stiffness but also reduced the mass to 44.4% of the initial model. From the studies and applications of generative design, it can be see that it can automatically generate a large number of design schemes in a short period, which is convenient for designers as it allows them to choose the most appropriate scheme. Additionally, the results of generative design have a good mechanical property and light mass. Consequently, if the generative design is utilized in the design of cross joints, it is expected to improve the efficiency of the joint design optimization.

On the other hand, using the casting process of pouring liquid steel into a mold in one piece, various joints with complex configurations can be produced, and overlapping welds at the intersecting parts can be avoided during welding [9]. Therefore, cross-cast-steel joints have been widely applied in engineering during the last few years. However, the conventional fabrication process of cast-steel joints is mandrel, sandbox, casting, and polishing [23], and therefore the manufacturing cycle is long. Meanwhile, a large number of manual operations lead to huge labor consumption, especially when the types and numbers of joints in an entire structure are substantial, as shown in Figure 1b. 3D printing is a transformative advanced digital manufacturing technology, developed in the past 30 years [24,25,26]. Differently from traditional fabrication methods (cutting, grinding, casting, forging, etc.), the technology cuts the whole model through a program into digital slices, automatically generates printing commands after sorting, and prints the digital slice layer by layer for rapid prototyping. Many experts and companies have applied 3D printing technology in the field of building structures. For example, in 2018, MX3D collaborated with AURP to presented the world’s first metal bridge manufactured with 3D printing technology at the Dutch design week [27]. Kanyilmaz et al. [28] utilized 3D printing technology to achieve a nature-inspired optimization design of steel tubular joints. Du et al. [29] conducted an integrated study of topology optimization and additive manufacturing on a cable-dome bearing joint and obtained the result that when the density support was 15%, the surface of the manufactured joint was relatively smooth. Compared with conventional fabrication technologies, 3D printing can manufacture complex structural models, effectually simplifying production processes and shortening manufacturing cycles [30]. In addition, 3D printing technology has unique advantages in the integration of design and manufacturing and effectively communicates the connection between the digital world and the physical world. Currently, 3D printing combined with generative design has been tested in other fields, such as the redesign of a seatbelt bracket by General Motors and the design of cabin partitions by the European Aeronautics and Astronautics Corporation for Airbus [6]. From these studies, it can be seen that generative design can make full use of the design space provided by 3D printing technology, and 3D printing can ensure the manufacturability of generative results. Therefore, utilizing generative design and 3D printing will help to realize the integrated process of design and fabrication, and then effectively solve the problems of excessive mass, insufficient geometric optimization, and long production time for cross joints in practical engineering.

This work is presented as follows: In Section 2, the method and mathematical model of generative design are established and elaborated. In Section 3, reasonable configurations of cross joints under axial force are obtained by the generative design method; three representative joint models are then selected for further numerical analysis and static behavior comparison to verify the advantages of generative design. In Section 4, 3D printing technology is used to manufacture generative joints.

## 2. Method and Mathematical Model of Generative Design

### 2.1. Method

Generative design is a hybridization of the design and optimization method, usually integrated into a computation-intensive machine learning algorithm executed on powerful cloud computing technology [13,31,32,33,34]. This method integrates objective engineering requirements into subjective design, and through the paradigm of simulating natural growth and evolution, unlimited new viable design schemes can be created in a comparatively short time [35,36,37,38,39]. Generative design only requires clarifying the design conditions and target performance and does not depend on the initial structural geometry [19,40,41]. Generative programs relating to the intelligent distribution of the material in the cloud can automatically generate more innovative and feasible design alternatives that have a lighter mass and better properties. In addition, the relevant data of each generative scheme is fed back in real-time into the cloud to evaluate and screen out the best structural configuration suitable for practical engineering.

**Figure 2:**
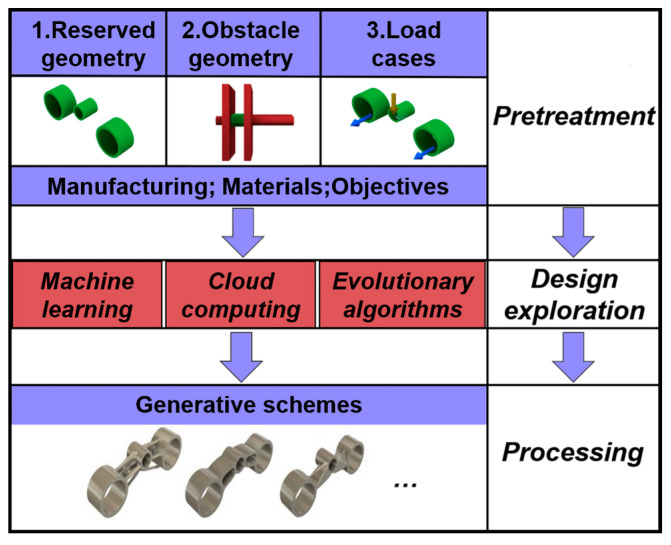
The process of generative design.

In the whole design process, the generative program can automatically complete the various contents, effectively prevent the large number of repeated calculations and adjustments required by traditional methods, and markedly upgrade the efficiency and intelligence of the structural design [42,43,44]. To enhance intuitive understanding, this work explains the idiographic operation process of the generative design method, mainly including pretreatment, design exploration, and processing, as shown in Figure 2. The first is the pretreatment of generative design, including defining the reserved geometry and obstacle geometry of structures, arranging loads and imposing constraints on the corresponding components, choosing manufacturing methods and materials, and setting generation objectives. Generative iteration and model optimization are then performed based on machine learning, evolutionary algorithms, and cloud computing. Finally, the designer can select any generative scheme for output.

### 2.2. Mathematical Model

Generative design starts from a series of problem solutions and evaluates multiple solutions in the design space to maximize the quality of the generated model. To attain more diversified structural design alternatives, this study combines space-filling criterion and non-cohesion criterion based on the generative design method. The space-filling design is normally applied in computer experiments, and the non-cohesion criterion is a potent rule to ensure that there will be no interference between design schemes [22,45]. The mathematical model of generative design is as follows:

(1) $$x_{w}=\left\{x_{\left(w,k\right)},k=1,2,\ldots n\right\}\in Z\subseteq R$$

(2) $$Z=\left\{x_{w}^{l}\leq x_{\left(w,k\right)}\leq x_{w}^{u},\forall k\in \left\{1,2,\ldots n\right\}\right\}$$

Design space Z is composed of a series of solutions w, represented by $x_{w}$. Z is a subset of R, which is constrained by the lower limit $x_{w}^{l}$ and upper limit $x_{w}^{u}$.

The generative design objectives are to explore Z and generate a set composed of N different schemes. N is a custom parameter, and each specific position in Z represents each project in m. In order to attain the set m, the space-filling criterion $G\left(m\right)$ is introduced to solve the project (Equation (3)).

(3) $$G\left(m\right)=\overset{N-1}{\underset{p=1}{\sum }}\overset{N}{\underset{q=p+1}{\sum }}\frac{1}{E{\left(x_{p},x_{q}\right)}^{2}}$$

(4) $$E\left(x_{p},x_{q}\right)={\left(\overset{n}{\underset{k=1}{\sum }}{\left(x_{p,k}-x_{q,k}\right)}^{2}\right)}^{\frac{1}{2}}$$

$E\left(x_{p},x_{q}\right)$ is the Euclidean distance between the design values *p* and *q*. According to the maximum distance criterion, the minimum value of $G\left(m\right)$ is conducive to the equidistribution of N schemes in Z.

In high-dimensional design space, Audze space-filling criteria will place different alternatives on the boundary of design space, which is not desirable [46]. Hence, it is necessary to introduce a non-cohesion criterion, which divides each dimension of Z into N intervals and guarantees that the two schemes do not interfere with each other. The criterion utilizes Equation (5) to merge the retrieval process, and Equation (5) calculates the number of intervals shared by N schemes. On the basis of the parameter θ, the adjusted weight of $H\left(m\right)$ can create a complete scheme of minimization.

(5) $$H\left(m\right)=\theta \times \overset{N-1}{\underset{p=1}{\sum }}\overset{N}{\underset{q=p+1}{\sum }}o\left(y_{p},y_{q}\right)$$

(6) $$o\left(y_{p},y_{q}\right)=\overset{n}{\underset{j=1}{\sum }}f\left(y_{p,k},y_{q,k}\right)$$

(7) $$f\left(y_{p,k},y_{q,k}\right)=\left\{\begin{array}{c} 1,\;y_{p,k}=y_{q,k}\\ 0,\;y_{p,k}\neq y_{q,k} \end{array}\right.$$

In Equation (5), $o\left(y_{p},y_{q}\right)$ is the interval number shared between the design values *p* and *q*; $y_{p}$ and $y_{q}$ are the discrete representations of $x_{p}$ and $x_{q}$, respectively. To solve the k−th geometric parameter $x_{i,k}$ of the i−th design scheme, the generative design first divides the range between the lower limit $x_{i,l}^{k}$ and the upper limit $x_{i,u}^{k}$ into N intervals $\left[x_{i,l}^{k}=x_{i,l}^{1},x_{i,l}^{2},\ldots x_{i,l}^{N}=x_{p,k}^{u}\right]$, and then assigns the integer coordinate t to $y_{i,k}$, where $y_{i,k}$ is the discrete value of $x_{i,k}$, as shown in (Equation (8)).

(8) $$\forall t=\left\{1,2,\ldots N\right\},\left(x_{i,l}^{t}\leq x_{i,k}\leq x_{i,l}^{t+1}\right)\to \left(y_{i,k}=t\right)$$

(9) $$F\left(m\right)=\overset{N-1}{\underset{p=1}{\sum }}\overset{N}{\underset{q=p+1}{\sum }}\frac{1}{E{\left(X_{p},X_{q}\right)}^{2}}+\theta \times \overset{N-1}{\underset{p=1}{\sum }}\overset{N}{\underset{q=p+1}{\sum }}o\left(y_{p},y_{q}\right)$$

In a generative design, each iteration is accomplished by performing N sub iterations. After the objective function $F\left(m\right)$ converges, the generative algorithm returns the m optimal set of N. The generative design goal for cross joints in this study is to provide maximum bearing capacity while utilizing the least amount of material. Because stiffness is the most direct characteristic value of bearing capacity [47], the maximum stiffness is selected as the design objective. The volume V and compliance C are simultaneously minimized, and the optimization problem is expressed as follows:

(10) $$\min:\left\{\begin{array}{l} C=\frac{1}{2}U^{T}KU\\ L=(V/V_{\max})\times 100\% \end{array}\right.$$

where U is the displacement vector of the cross joints, K is the global stiffness matrix, V is the volume of each iteration of joints, and $V_{\max}$ is the volume of the whole design space.

## 3. Generative Design of the Cross Joint

### 3.1. Pretreatment of Generative Design

**Figure 3:**
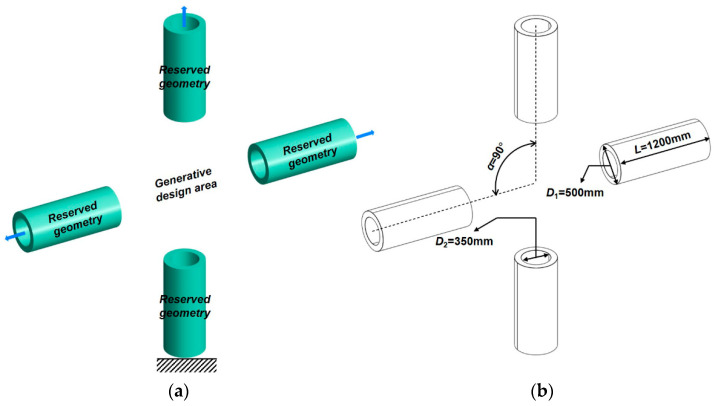
The initial model of a cross joint for the generative design: (**a**) initial joint model, (**b**) geometric properties.

The generative design does not depend on the initial structural model and only requires specifying the reserved geometry and obstacle geometry of the structure. Reserved geometry refers to the geometric features that need to be included in the final structural shape. Obstacle geometry refers to the geometric features that need to be excluded. In this study, four-branch pipes are selected as the reserved geometry; the middle part is the generative design area, which is used to obtain the initial cross joint model for generative design, as shown in Figure 3. The model is then imported into the calculation program for generative design.

Subsequently, the constraint and load are applied to the initial joint, as shown in Figure 3a. Choosing the bottom surface of the initial model as the constraint surface, the constraint is regarded as a fixed end. The axial tension of the 645 kN load is arranged on the remaining three pipes, respectively. The design objective is maximum stiffness. Material properties are defined and listed in Table 1.

### 3.2. Analysis of Generative Joints

**Figure 4:**
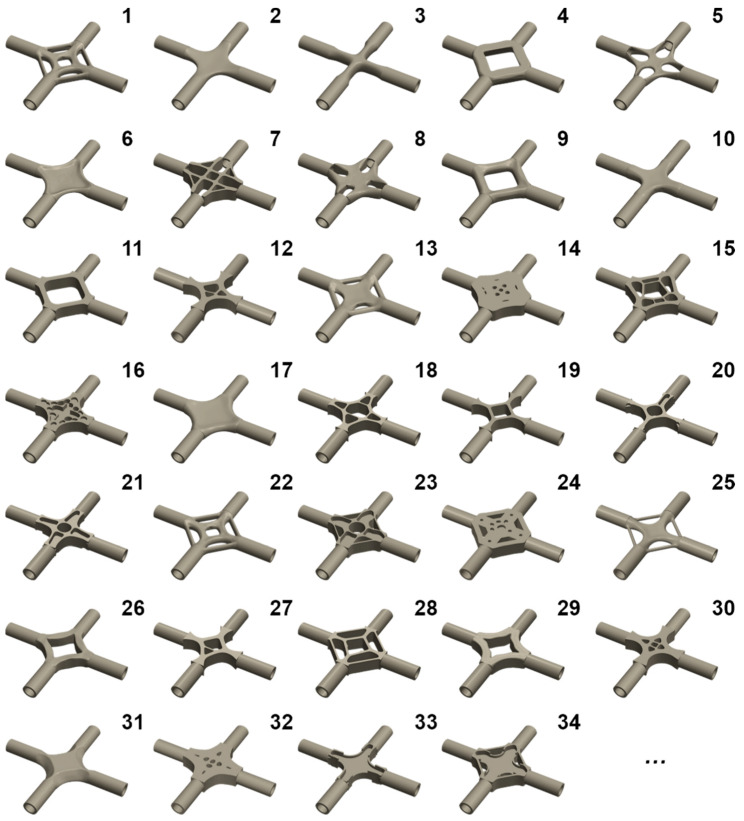
Partial generative design results of the cross joint.

After checking the input parameters, the cloud platform is set for iterative calculations to generate multiple design schemes based on the structural performance requirements. The generative design leverages the powerful operational capacity of cloud computing to simultaneously design thousands of cross joint models in a short period. This study lists only some particular design schemes generated automatically by the generative design and codes them, as shown in Figure 4.

It is easy to see from Figure 4 that the configurations of generative joints are varied and innovative. Many joint models exhibit unique geometric features.

- There is no material in the middle of generative joints 4 and 9, and each branch is connected by a hollowed-out cube;
- The transition between each branch and the generative design area of generative joint 5 is smooth;
- The generated part of generative joint 7 looks like a shape that has been cut by a tool and has an excellent visual effect;
- Generative joints 26 and 29 seemingly come from the same iterative design, and their branches are united in a crescent shape, which is symmetrical as a whole. It can also be seen that generative joint 26 has a higher degree of evolution than 29, and the model is lighter and simpler.

The above joints embody the powerful model generation ability of the generative design method. Furthermore, the generative joint configurations are different from the cross joint shapes in engineering applications, which is beyond the scope of imagination based on the engineers’ experience. In summary, the generative design method can automatically generate multiple neoteric structural configurations under the given conditions. The physical index values of each generated result also are obtained by the generative algorithm, such as volume, mass, maximum equivalent stress, and maximum displacement, which provides convenience for designers to choose the most appropriate scheme.

**Figure 5:**
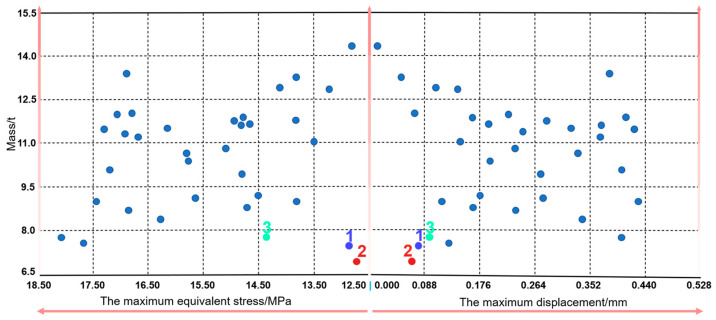
Scatter diagram of the generative cross joints.

To further verify the mechanical performance of the generative cross joints, the general software (HyperWorks 2021) was used to carry out linear FE analysis. Due to the large number of generative joints, the workload of one-by-one numerical verification is substantial. Therefore, this work comprehensively considers the practical application requirements and cost indexes of the cross joint and selects the representative joints with lighter mass for further numerical analysis. To choose representative joints more intuitively, this paper integrates the data values obtained by the generative algorithm into a scatter plot, as shown in Figure 5. It is not difficult to see from Figure 5 that the mass and mechanical properties of generative joints 1, 2, and 3 are the best. Consequently, these three joints were selected as representative generative joints and are marked in Figure 5.

### 3.3. Static Behaviors of the Representative Joints

**Figure 6:**
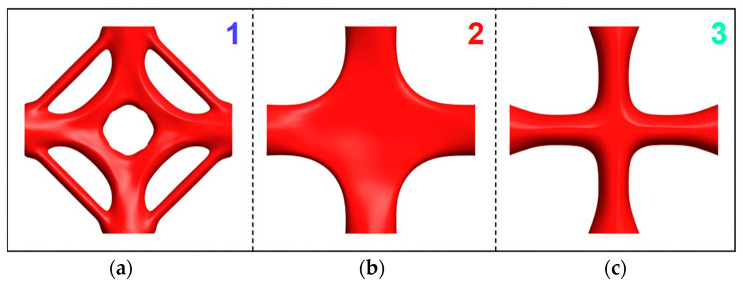
The details of representative generative joints: (**a**) generative joint 1, (**b**) generative joint 2, (**c**) generative joint 3.

The detailed characteristics of the generative design area for the representative joints are depicted in Figure 6. The generative joints have a novel shape, the generative part has outstanding compatibility with the tetrahedron, and the transition is smooth.

**Figure 7:**
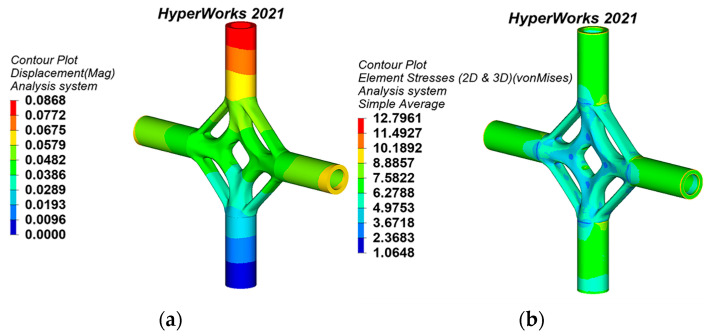
Static analysis results of generative joint 1: (**a**) displacement nephogram, (**b**) equivalent stress nephogram.

**Figure 8:**
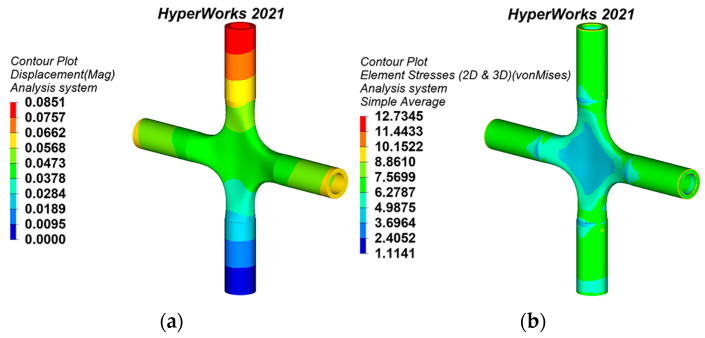
Static analysis results of generative joint 2: (**a**) displacement nephogram, (**b**) equivalent stress nephogram.

**Figure 9:**
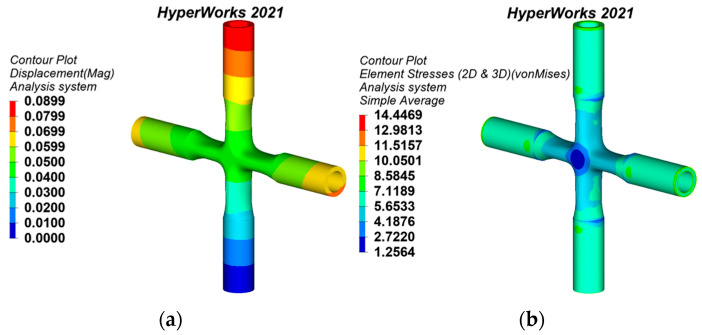
Static analysis results of generative joint 3: (**a**) displacement nephogram, (**b**) equivalent stress nephogram.

The joint models are imported into the software HyperWorks 2021 in step format. The material PSOLID entity attributes are created and provided in HyperMesh. In this study, to ensure the quality of mesh generation, 2D mesh generation is first performed on the joint model, and the 2D mesh is checked in detail. The 2D mesh is then converted to a 3D mesh through Tetra mesh, the quality of the 3D mesh is checked, and any failed mesh is modified. Finally, the FE model of the cross joint is attained. The load and constraint are consistent with the original model of generative design. All nodes on the bottom-edge surface of the lower pipe are selected on the joint FE model to constrain the translational and rotational degrees of freedom in the X, Y, and Z axes. An axial tension of 645 kN is arranged on the top-edge surface of the remaining three branch pipes. Because the cast steel material has obvious plasticity, the ideal elastoplastic model is adopted, obeying the von Mises yield criterion. The static analysis results of displacement and the equivalent stress of the generative joints are shown in Figure 7, Figure 8 and Figure 9.

The static analysis results show that the stress distribution of generative joints is uniform, and the displacement and stress distribution of each joint are identical, which indicates the rationality of the generative results. To further analyze the practical optimized design effect of the generative cross joints, the static behaviors are compared with those of traditional flat polygon joints and topology-optimized joints.

### 3.4. Comparison Analysis with Other Joints

#### 3.4.1. Static Behaviors of the Flat Polygon Cross Joint

**Figure 10:**
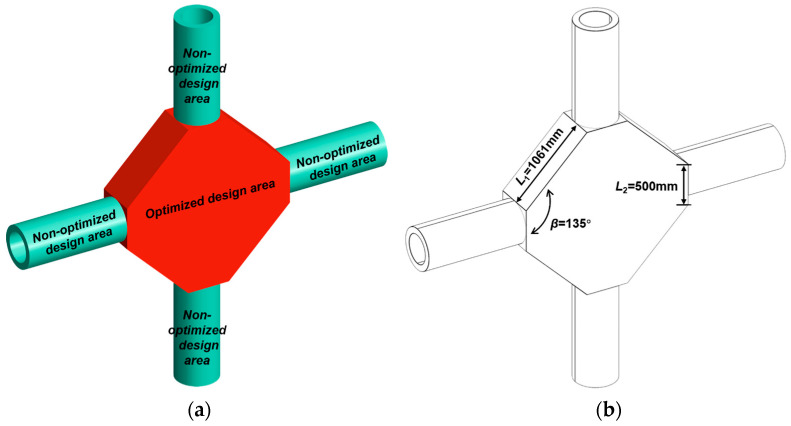
The flat polygon cross joint: (**a**) model, (**b**) geometric features.

The flat polygon is a commonly used connection for cross joints, so it has been selected to compare its mechanical performance with the generative joints [5]. The flat polygon cross joint model and its geometric characteristics are shown in Figure 10; its branch size and material properties are the same as the initial cross joint model designed by generative design. The flat polygon cross joint is taken as the original model of the topology-optimized joints.

**Figure 11:**
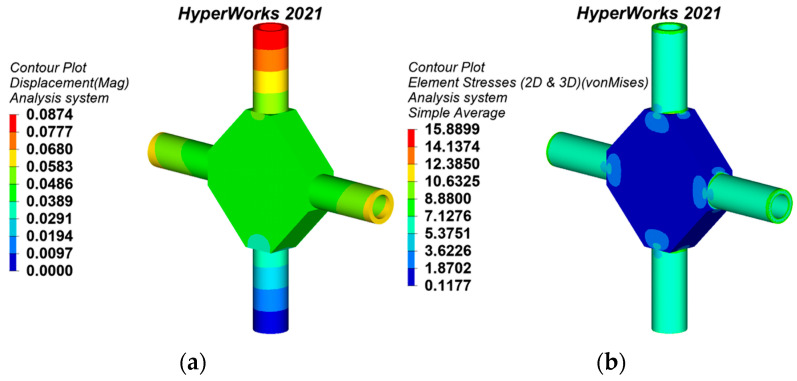
Static analysis results of the flat polygon cross joint: (**a**) displacement nephogram, (**b**) equivalent stress nephogram.

The mass of the flat polygon cross joint is 15.062 t. Under the same working conditions as the generative joints, the static analysis results are shown in Figure 11. The maximum displacement is 0.0874 mm, which is located at the top of the upper part of the pipe. The maximum equivalent stress is 15.8899 MPa, which is located outside each branch pipe and the connection of the flat polygon of the joint.

#### 3.4.2. Static Behaviors of the Traditional Topology-Optimized Joint

**Figure 12:**
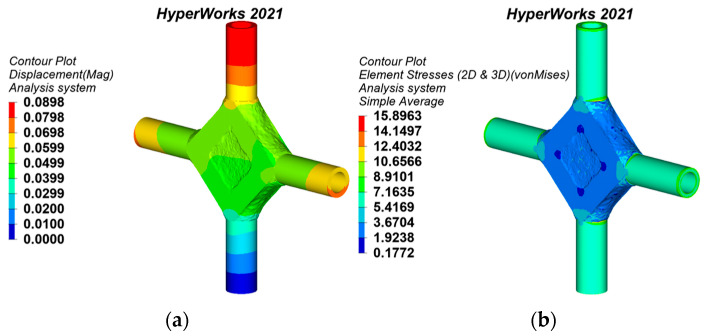
Static analysis results of the traditional topology-optimized joint: (**a**) displacement nephogram, (**b**) equivalent stress nephogram.

According to the research status of joint design, the topology optimization method can minimize the weight while ensuring the excellent mechanical performance of joints [4,9]. Hence, the topology-optimized joint is elected to compare its static behavior with the generative joints. The static analysis results are shown in Figure 12. The mass of the topology-optimized joint is 10.675 t. The maximum displacement is 0.0898 mm, which is located at the top of the upper pipe. The maximum equivalent stress is 15.8963 MPa, which is located outside the intersection of each branch and the flat polygon of the joint.

#### 3.4.3. Static Behaviors of Substructure Topology-Optimized Joints

Based on our recent research for the optimization design of cross joints, topology optimization using traditional substructure and bionic substructure partitioning methods has better overall performance than the joint model obtained by conventional topology optimization, and the related achievements have been published previously in journals [4]. To fully verify the actual optimization level of the generative design, the traditional substructure topology-optimized joint and the bionic-based substructure topology-optimized joint are selected to compare their static behavior with the generative joints.

**Figure 13:**
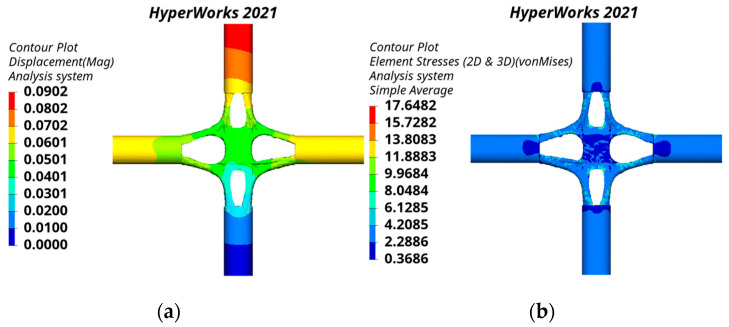
Static analysis results of the traditional substructure topology-optimized joint: (**a**) displacement nephogram, (**b**) equivalent stress nephogram.

The static analysis results of the cross joint obtained by the traditional substructure topology optimization are shown in Figure 13. The mass of the traditional substructure topology-optimized joint is 9.930 t. The joint displacement and stress are symmetrically distributed. The maximum displacement is 0.0902 mm, which is located at the top of the branch pipe. The maximum equivalent stress is 17.6482 MPa, which is located near the junction of the branch pipe and main pipe.

**Figure 14:**
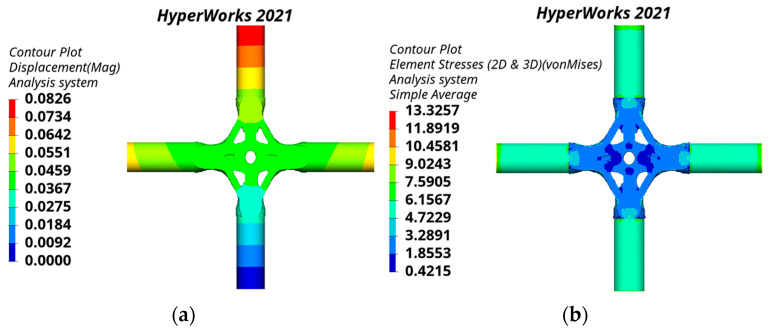
Static analysis results of the bionic-based substructure topology-optimized joint: (**a**) displacement nephogram, (**b**) equivalent stress nephogram.

The static analysis results of the joint obtained by the bionic-based substructure topology optimization method are shown in Figure 14. The joint has a mass of 9.870 t. The maximum displacement and the maximum equivalent stress of the joint are the same as the distribution position of the joint obtained by the traditional substructure topology optimization, and the values are 0.0826 mm and 13.3257 MPa.

#### 3.4.4. Comparison Analysis and Discussion

To facilitate the comparative analysis for the mechanical performances of the above joints, the mass, maximum displacement, and equivalent stress are summarized in Table 2. The flat polygon cross joint model is applied as a reference object for comparison. Negative values indicate a decrease, while positive values indicate an increase.

It can be seen from Table 2 that the static behaviors of the topology-optimized joints and the generative joints have been improved in comparison to the flat polygon cross joint model. In terms of joint maximum equivalent stress, generative joint 2 has the best manifestation, which is reduced by 19.86%. In addition, it can be seen from Figure 8b that the color bar of the equivalent stress nephogram of generative joint 2 is not much different, and the stress distribution is more uniform than that of other joint models. In terms of joint maximum displacement, the bionic-based substructure topology-optimized joint has the best manifestation, which is decreased by 5.49%. In terms of joint mass, compared with the flat polygon cross joint model, the mass of the generative joints and the topology-optimized joints has been abated, among which the weight of generative joint 2 is reduced by 53.92%, and the effect is the most prominent. Therefore, generative joint 2 is optimal; not only is mass minimized, but the mechanical properties are also greatly improved. Furthermore, the topology-optimized joint surface is rough, while the generative joints are smooth and attractive, which is more in line with the needs of architectural structure aesthetics. In summary, the joints obtained by generative design have the most balanced stress distribution and best static behaviors, which notably improves the design optimization effect of the cross joints.

## 4. Manufacturing of Generative Joints

In recent years, 3D printing has become an emerging industrial intelligent manufacturing technology. Different from the traditional manufacturing method, the technology converts the concept of subtracting materials into adding materials [48]. Based on digital model files, it utilizes powdered adhesive materials to construct objects by stacking them layer by layer [49,50,51]. At present, the main 3D printing technologies are fused deposition modeling (FDM), selective laser melting (SLM), laminated object manufacturing (LOM), stereo lithography appearance (SLA), and wire and arc additive manufacturing (WAAM) [47,52].

**Figure 15:**
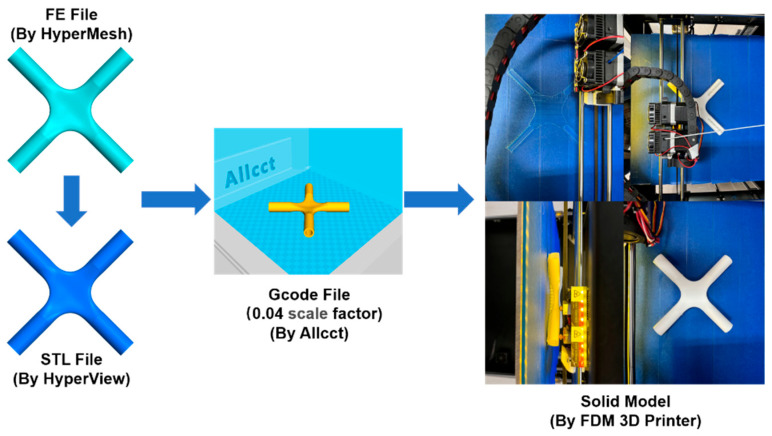
3D printing technology of the generative joint.

Limited to experimental equipment and by cost, this paper utilizes FDM technology to manufacture scale models of the generative joints with polylactic acid (PLA) plastic as materials to verify the feasibility of 3D printing technology in restoring joint features and enhance the intuitive understanding of generative joints. FDM technology works by squeezing materials heated at high temperatures through a nozzle to build a physical model layer by layer [53,54,55]. The production process is characterized by converting the FE file of generative joints into the stereolithography (STL) file and then reading it into the slicing software to generate the 3D printing commands, which are finally exported to the 3D printer of the Advanced Design and Intelligent Manufacturing Laboratory of Henan University for additive manufacturing. The 3D printing process is shown in Figure 15.

**Figure 16:**
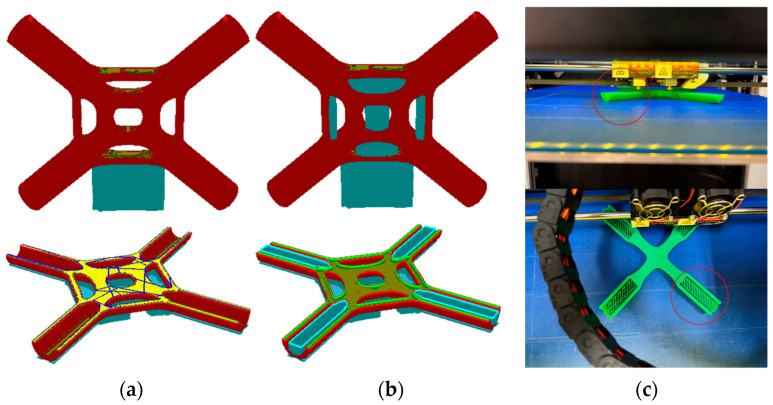
Support of 3D printing: (**a**) local support, (**b**) global support, (**c**) support details.

After many experiments, it was confirmed that the reasonable setting of some parameters is the key to attaining high precision joint models, such as printing temperature and speed, support, and nozzle diameter. The printing temperature is the most important parameter affecting the accuracy and mechanical performance of the finished model during the FDM printing process. The temperature of the printer nozzle should be kept 4~6% higher than the melting temperature of the printing material. The printing speed must be controlled within an appropriate range. If the printing speed is too quick, the underlying material will not have enough time to solidify. If the printing speed is too slow, the cooling time of the material will be longer, which may cause the adhesion between adjacent layers to be weakened, along with the layers’ uneven shrinkage. In addition, this study also lists the support type influence on the printed product. Support is mainly divided into no support, local support, and global support. Figure 16a,b shows the differences between local support and global support under different printing angles; the local support is only established in the key feature parts of the model, and the global support is arranged in all of the suspended parts. Therefore, selecting the appropriate printing angle and support type is of great significance to the performance and cost of the printed product. In this paper, the generative joint is parallel to the printing platform, and it is printed with a global support. Details of the support material are shown in Figure 16c. The 3D printing manufacturing parameters of the joint model are shown in Table 3.

**Figure 17:**
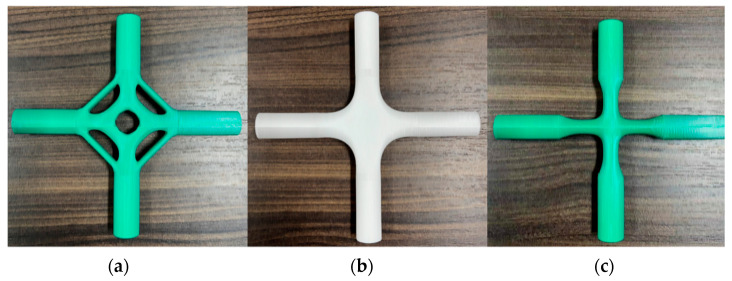
Solid models printed by the FDM 3D printer: (**a**) generative joint 1, (**b**) generative joint 2, (**c**) generative joint 3.

3D printing can highly reproduce the details of the joint model, which is very difficult to manufacture by traditional fabrication processes. It can be seen from Figure 17 that the generative joints are symmetrical, smooth, and balanced as a whole, and the connection between the generative design area and the main branch pipe is dense and glossy. Although the finished product made of PLA material cannot be applied directly to engineering practice, it provides a new idea for the manufacture of complex-shaped joints in the future. For example, 3D printing is used to produce the mold of complex cast-steel joints; digital processing can ensure the precision of the mold, decrease the local defects during casting, and enhance the manufacturing speed. Compared with traditional wooden molds, this method lowers the cost of a single mold. In addition, this method can also guarantee the actual performance of the joints. Although 3D printing technology has undergone unprecedented development in recent years, there are still many defects, such as the actual performance of 3D printed products. The actual failure mode of the printed model differs from the results of the finite element analysis due to many factors such as deposition angle, environmental condition of the 3D printer, and the polymer used to print. Therefore, the molds are produced using 3D printing technology, and then models are manufactured using the casting process. This not only shortens the production cycle but also ensures the mechanical properties of the joints.

## 5. Conclusions

In this work, a pioneering idea using the generative design method is successfully realized to attain a reasonable configuration for cross joints, which are consecutively manufactured using 3D printing technology. The main conclusions are as follows:

(1) The generative design method has powerful model generation ability and innovation ability. Generative design can automatically generate multiple new cross joint configurations. Most of the generative joints have novel shapes that are beyond the imagination of designers based purely on experiences. Furthermore, it is not always possible to manufacture joints generated by generative design because of the production limitations, molds, etc.

(2) Generative design can enhance the design optimization level of cross joints. The numerical simulation results indicate that the generative joints have a lighter mass and better mechanical performance. Moreover, the boundary and constraint conditions of the generative design method are straightforward, and the whole design process is more automatic and intelligent.

(3) Generative cross joints have excellent manufacturability and aesthetics. In this paper, 3D printing technology is applied to produce a reduced-scale model of the generative joints. The solid model shows that the joint formation effect is excellent, and the surface is smooth.

(4) The development of 3D printing technology ameliorates the manufacturing technology of cross joints. High precision joints can be produced using 3D printing.

(5) The combination of generative design and 3D printing is an efficient way to realize the integrated process of the design and manufacture of cross joints.

## Figures and Tables

**Table 1:** Material properties of the cross joint.

Parameters	Values
Density (ton/mm^3^)	7.85 × 10^−9^
Poisson’s ratio	0.3
Shear modulus (MPa)	8.077 × 10^4^
Elastic modulus (MPa)	2.10 × 10^5^

**Table 2:** Comparison of the generative joints and other joints.

Joint	Mass (t)	Compared to Flat Polygon Cross Joint (%)	Maximum Displacement (mm)	Compared to Flat Polygon Cross Joint (%)	Maximum Equivalent Stress (MPa)	Compared to Flat Polygon Cross Joint (%)
flat polygon cross joint	15.062		0.0874		15.8899	
traditional topology-optimized joint	10.675	−29.13	0.0898	2.75	15.8963	0.04
traditional substructure topology-optimized joint	9.930	−34.07	0.0902	3.20	17.6482	11.07
bionic-based substructure topology-optimized joint	9.870	−34.47	0.0826	−5.49	13.3257	−16.14
generative joint 1	7.508	−50.15	0.0868	−0.69	12.7961	−19.47
generative joint 2	6.940	−53.92	0.0851	−2.63	12.7345	−19.86
generative joint 3	7.829	−48.02	0.0899	2.86	14.4469	−9.08

**Table 3:** Summary of the 3D printing parameters.

Layer Thickness (mm)	Infill Density	Temperature (°C)	Nozzle	Speed (mm/s)	Support Mode
0.2	20%	210	0.4	40	All support

## Data Availability

Some or all data, models, or codes that support the findings of this study are available from the corresponding author upon reasonable request.
